# Genetic diversity in the plasticity zone and the presence of the chlamydial plasmid differentiates *Chlamydia pecorum* strains from pigs, sheep, cattle, and koalas

**DOI:** 10.1186/s12864-015-2053-8

**Published:** 2015-11-04

**Authors:** Martina Jelocnik, Nathan L. Bachmann, Bernhard Kaltenboeck, Courtney Waugh, Lucy Woolford, K. Natasha Speight, Amber Gillett, Damien P. Higgins, Cheyne Flanagan, Garry S. A. Myers, Peter Timms, Adam Polkinghorne

**Affiliations:** Faculty of Science, Health, Education and Engineering, University of the Sunshine Coast, Sippy Downs, QLD 4558 Australia; Department of Pathobiology, Auburn University, Auburn, AL USA; School of Animal and Veterinary Sciences, University of Adelaide, Roseworthy, South Australia 5371 Australia; Australia Zoo Wildlife Hospital, Beerwah, QLD 4519 Australia; Faculty of Veterinary Science, The University of Sydney, New South Wales, 2006 Australia; Port Macquarie Koala Hospital, Port Macquarie, NSW 2444 Australia; Institute for Genome Sciences, University of Maryland School of Medicine, Baltimore, MD USA

**Keywords:** *C. pecorum* plasmid, Porcine hosts, *C. pecorum* comparative genomics, Cytotoxin gene, Phylogenetic relationships

## Abstract

**Background:**

*Chlamydia pecorum* is a globally recognised pathogen of livestock and koalas. To date, comparative genomics of *C. pecorum* strains from sheep, cattle and koalas has revealed that only single nucleotide polymorphisms (SNPs) and a limited number of pseudogenes appear to contribute to the genetic diversity of this pathogen. No chlamydial plasmid has been detected in these strains despite its ubiquitous presence in almost all other chlamydial species. Genomic analyses have not previously included *C. pecorum* from porcine hosts. We sequenced the genome of three *C. pecorum* isolates from pigs with differing pathologies in order to re-evaluate the genetic differences and to update the phylogenetic relationships between *C. pecorum* from each of the hosts.

**Methods:**

Whole genome sequences for the three porcine *C. pecorum* isolates (L1, L17 and L71) were acquired using *C. pecorum*-specific sequence capture probes with culture-independent methods, and assembled in CLC Genomics Workbench. The pairwise comparative genomic analyses of 16 pig, sheep, cattle and koala *C. pecorum* genomes were performed using several bioinformatics platforms, while the phylogenetic analyses of the core *C. pecorum* genomes were performed with predicted recombination regions removed. Following the detection of a *C. pecorum* plasmid, a newly developed *C. pecorum*-specific plasmid PCR screening assay was used to evaluate the plasmid distribution in 227 *C. pecorum* samples from pig, sheep, cattle and koala hosts.

**Results:**

Three porcine *C. pecorum* genomes were sequenced using *C. pecorum*-specific sequence capture probes with culture-independent methods. Comparative genomics of the newly sequenced porcine *C. pecorum* genomes revealed an increased average number of SNP differences (~11 500) between porcine and sheep, cattle, and koala *C. pecorum* strains, compared to previous *C. pecorum* genome analyses. We also identified a third copy of the chlamydial cytotoxin gene, found only in porcine *C. pecorum* isolates. Phylogenetic analyses clustered porcine isolates into a distinct clade, highlighting the polyphyletic origin of *C. pecorum* in livestock.

Most surprising, we also discovered a plasmid in the porcine *C. pecorum* genome. Using this novel *C. pecorum* plasmid (p*Cpec*) sequence, a) we developed a p*Cpec* screening assay to evaluate the plasmid distribution in *C. pecorum* from different hosts; and b) to characterise the p*Cpec* sequences from available previously sequenced *C. pecorum* genome data. p*Cpec* screening showed that the p*Cpec* is common in all hosts of *C. pecorum*, however not all *C. pecorum* strains carry p*Cpec*.

**Conclusions:**

This study provides further insight into the complexity of *C. pecorum* epidemiology and novel genomic regions that may be linked to host specificity. *C. pecorum* plasmid characterisation may aid in improving our understanding of *C. pecorum* pathogenesis across the variety of host species this animal pathogen infects*.*

**Electronic supplementary material:**

The online version of this article (doi:10.1186/s12864-015-2053-8) contains supplementary material, which is available to authorized users.

## Background

*Chlamydia pecorum* is a globally distributed animal pathogen of domesticated and wild ruminants, and the iconic native Australian marsupial, the koala. Expansion of the number of available whole genome sequences of *C. pecorum* from sheep, cattle, and koala hosts has provided insights into its lifestyle, associated virulence factors, and evolution [1–3]. The genetic differences that may influence host specificity and/or pathogenicity in *C. pecorum* remain largely unknown, however, with only (i) single nucleotide polymorphisms (SNPs); (ii) a limited number (four to five) of pseudogenes; and (iii) variable numbers of tandem repeats in certain *C. pecorum* genes (eg. ORF663 and *Inc*A genes), differentiating strains from different hosts or associated with different diseases [1–5].

In other chlamydial species, the almost ubiquitous non-integrative chlamydial plasmid [6, 7] has been recognised as a major chlamydial virulence factor [8, 9]. This plasmid also has value as a target for molecular typing [10] and as an immunogenic vaccine candidate both for humans and animals such as ducks, pigeons, cats and pigs [11, 12]. Two studies have previously detected a *C. pecorum* plasmid in isolates from three koalas and one bovid [13, 14], but further evidence for a *C. pecorum* plasmid has remained absent despite the growing number of genomics studies for this species [1–3, 15].

Although *C. pecorum* infects a wide range of hosts, including some other Australian marsupials, European wild ruminants, and pigs, *C. pecorum* comparative genomics studies thus far have focused only on sheep, cattle, and koala strains [16–18]. Porcine *C. pecorum* infections are underestimated as a source of on-farm economic loss for producers, manifesting as a variety of diseases such as pneumonia, pleuritis, polyserositis, polyarthritis, conjunctivitis, and enteritis. As with other hosts, *C. pecorum* subclinical infections in pigs are also common [18–22]. In an effort to broaden our knowledge of the genetic diversity of *C. pecorum* in other hosts, in the present study, we sequenced three porcine *C. pecorum* genomes and compared them to the available sheep, cattle, and koala *C. pecorum* genomes. In doing so, we also provide the first detailed description of the genetic structure and distribution of the *C. pecorum* plasmid, a genetic feature associated with virulence in other chlamydial species.

## Methods

### Descriptions of pig *C. pecorum* isolates, clinical samples and other *C. pecorum* strains used in this study

The three *C. pecorum* strains L1, L17 and L71 isolated from pigs presenting with pneumonia (L1 and L17) and polyarthritis (L71) were utilised for whole genome sequencing and plasmid analyses in the present study. These strains were one of the first pig chlamydial strains, isolated together with many others in 1969 in Austria, from pigs presenting with chlamydiosis during a mass chlamydial outbreak [20, 21].

Plasmid screening was performed on a large collection of (i) previously tested *C. pecorum* PCR positive clinical samples from sheep, cattle and koalas (Additional file 6: Table S4); (ii) and a small collection of cultured *C. pecorum* isolates from a variety of hosts (Additional file 4: Table S3); (iii) additional previously untested but *C. pecorum* PCR positive sheep and koala samples identified in this study (Additional file 6: Table S4). This latter testing, combined with our previous screening for *C. pecorum* DNA in clinical samples, resulted in (i) 89 *C. pecorum* PCR positive ocular and urogenital swab samples from 53 koalas from different populations across Australia; (ii) 83 ocular, joint, vaginal and rectal swabs collected from 41 sheep from nine different flocks from the Central NSW region in Australia [23, 24]; and (iii) ocular, nose, internal organs, and rectal samples (*n* = 9) from four cattle from two herds, one previously described from Western Australia (WA) [25] and a second from Central NSW (Additional file 6: Table S4). Cultured *C. pecorum* isolates from pigs, sheep, cattle, and koalas screened are listed in Additional file 4: Table S3.

### Genomic DNA extraction and *C. pecorum*-specific qPCR screen

New sheep, cattle and koala clinical swab samples used in this study were processed as previously described [23]. The cultures of three *C. pecorum* porcine isolates L1, L17 and L71 were propagated in chicken embryos and purified, as previously described [22], while the cultures of 14 koala isolates were propagated in Hep-2 and/or McCoy cells and semi-purified using a probe sonication and density gradient centrifugation, as previously described [1]. DNA extraction for both samples and cultures was performed using the QIAmp DNA mini kit (Qiagen), as per the manufacturer’s instructions. *C. pecorum* infectious load and/or presence were determined using a *C. pecorum* species-specific qPCR assay targeting 202 bp of 16S rDNA [5]. Samples with < 50 copies *C. pecorum* 16S rDNA were considered negative. DNA concentration for each sample was also measured in duplicates on a Nanodrop. Additionally, 1 μl of extracted, screened and quantified gDNA of three porcine (L1, L17 L71) *C. pecorum* samples were run on a 1 % TBE agarose gel to confirm high molecular weight DNA prior to whole genome sequencing.

### Sequence capture, whole genome sequencing and assembly

Sequence capture was performed on total DNA extracted from *C. pecorum* L1, L17 and L71 cultures with the *C. pecorum* custom-designed probes (made by Agilent Technologies) [2], using a DNA hybridisation capture and amplification process based on the methods described elsewhere [26]. L1, L17 and L71 genomes were sequenced at the Institute for Genome Sciences (IGS), Baltimore, USA, using the Illumina HiSeq 2500 platform producing paired-end 101 base-pair reads. Read quality, *de novo* assembly and read-mapping of paired-end reads from L1, L17 and L71 was performed using CLC Genomics Workbench (CLC bio, Qiagen), after filtering and trimming for size and quality. The read mapping was visualised with BLAST ring image generator (BRIG) software [27]. After *de novo* assembly, L1, L17 and L71 contigs were blasted to confirm identity using BLASTn (Basic Local Alignment Search Tool from http://blast.ncbi.nlm.nih.gov/Blast.cgi) and ordered against complete *C. pecorum* E58 genome (accession number: CP002608) with progressive Mauve [28] to produce single genome scaffold. Genome annotations were done using RAST [29] and the IGS prokaryotic annotation pipeline, assisted by mapping onto the *C. pecorum* E58 type strain sequence, as previously described [15]. Number and distribution of SNPs was determined using the Probabilistic variant detection plug-in with default settings with L1, L17 and L71 reads mapped to a reference genome, as implemented in CLC genomics workbench. General descriptions and accession numbers of L1, L17 and L71 genomes were outlined in Table 1.

### Macroscopic comparative genomics and phylogenetic analyses using new porcine genomes

Alongside the newly sequenced three porcine *C. pecorum* genomes, we also used publicly available genome sequences of *C. pecorum* from: cattle encephalomyelitis E58 [15] (accession number: CP002608); sheep arthritis P787 (accession number CP004035.1), sheep fecal W73 (accession number CP004034.1), cattle metritis PV3056/3 (accession number CP004033.1) [3]; koala cystitis Mc/Marsbar (accession number CM002310.10) and DBDeUG (accession number CM002308.1), koala conjunctivitis IpTaLE (accession number CM002309.1), sheep polyarthritis IPA (VR629) (accession number CM002311.1) [1]; cattle encephalomyelitis NSW/Bov/SBE (accession number SRR1693788), sheep polyarthritis Mer/Ovi1/Jnt (accession number SRR1693791), sheep conjunctivitis Nar/S22/Rec (accession number SRR1693794), koalas with cystitis Gun/koa1/Ure (accession number SRR1693763) and SA/K2/UGT (accession number SRR1693792) [2] for whole genome comparative analyses.

Pairwise comparative genomic analyses were performed in ACT, the Artemis Comparison Tool [30] and Geneious 7.1.4 [31] using alignments generated with progressive Mauve [28]. Polymorphic loci and/or regions of interest in the genomes were extracted and further aligned with ClustalX [32]. DnaSP 5.0 [33] was used to analyse the level of sequences polymorphisms by: determining the ratio of non-synonymous to synonymous substitutions (d_n_/d_s_) (Jukes-Cantor corrected); calculating the number of polymorphic (segregating) sites and haplotype diversity. Further, if a polymorphism resulted in a non-synonymous amino acid change or premature STOP codon in the loci of interest in each of the analysed *C. pecorum* genome sequence, the translated sequence was further analysed in BLAST for comparison (http://blast.ncbi.nlm.nih.gov/Blast.cgi),UniProt (Universal Protein Resource from http://www.uniprot.org/) and Conserved Domains Database (CDD from http://www.ncbi.nlm.nih.gov/cdd) to assess protein functionality. Figures of the genomic regions of interest, such as the PZ was generated using Easyfig [34], based on tblastx comparison. Construction of phylogenetic relationships between the loci was performed using MrBayes [35] as implemented in Geneious 7.1.4.

In addition to the subset of 16 *C. pecorum* genomes used for comparative analyses, for phylogenetic analyses we have included: (i) sheep Nar/S22/RE (accession number SRR1693793); (ii) as well as the two secondary koala *C. pecorum* strains (Gun/koa1/Ure-ß, SA/K2/UGT-ß) identified in the same study [2]. Conserved regions were identified in 19 *C. pecorum* genomes using all-versus-all BLAST search. Syntenic regions were individually aligned using Muscle [36] and concatenated to produce a ~ 280 kbp core genome alignment. Poorly aligned regions were removed from the alignment using GBLOCKs with the minimum length of a block set to 5 and no gap positions were allowed. Recombination regions were predicted using Gubbins [37] and subsequently filtered from the alignment. A mid-point rooted phylogenetic tree was constructed from the genome alignment with PhyML 3.1 using the GTR model. Bootstrap values were calculated using 500 replicates.

### PCR amplification and phylogenetic analyses of *tox*C

During pairwise comparative genomic analyses, the presence of a third copy of the cytotoxin gene (*tox*C) in porcine L71 genome was identified. Primers (toxC For (5’ TCA GAG AGA AGC AGC TTA G 3’) and toxC Rev (5’ TTC TTG AGA AGT AAC ACT ACG 3’)) were designed to amplify a 644 bp fragment of the *tox*C gene in order to confirm the presence of this sequence. Using purified L71 genomic DNA as a template (and positive control), and purified *C. pecorum* koala MC/Marsbar and IpTaLe, cattle E58, porcine L1, L17 and sheep IPA genomic DNA as negative controls, conventional PCR assay was performed to amplify the 644 bp *tox*C fragment. PCR assays for 644 bp *tox*C fragment were prepared to a total reaction volume of 50 μl including 1X Amplitaq Gold® 360 Master Mix (Life Technologies, Victoria, Australia), 0.3 μM of forward and reverse primer each (Integrated DNA Technologies New South Wales, Australia) and 3 μl DNA template. The cycling conditions included an initial denaturation (10 min, 95 °C) followed by 35 cycles of denaturation (30s, 95 °C), annealing (30s, 53.5 °C) and extension (1 min, 72 °C), followed by a final extension (7 min, 72 °C). The amplicon was then purified and dideoxy sequenced (described in more detail in the following section below). After successful amplification and confirmation of the *tox*C fragment in L71 only, we tested the remaining eight porcine, seven cattle, two sheep (W73 and JP-1-751) and five koala *C. pecorum* isolates (Additional file 4: Table S3) for the presence of the *tox*C 644 bp fragment sequence.

Identification of the full length L71 *tox*C sequence was checked in BLAST and the midpoint rooted Bayesian phylogeny was constructed using alignment of all three cytotoxin (*tox* A, B and C) genes from a subset of 12 *C. pecorum* analysed in this study. The phylogenetic tree was constructed with MrBayes as implemented in Geneious 7.1.4, using the HKY + I model with four Markov Chain Monte Carlo (MCMC) chains with a 150 000 generations, sampled every 100 generations and with the first 10 000 trees were discarded as burn-in. *C. muridarum* Nigg (accession number: AE002160) three cytotoxin gene sequences were also included in the alignment for phylogenetic analyses, with the *C. muridarum* Nigg *tox* 3 used as an out-group.

### *C. pecorum* plasmid contig identification

The L1 7.5kbp plasmid contig was annotated with RAST [29], and further curated in Geneious 7.1.4. Additionally, p*Cpec* L1 sequence was also blasted against raw reads of *C. pecorum* porcine L17 and L71, sheep IPA, cattle NSW/Bov/SBE, and koala Mc/Marsbar, DBDeUG, IpTaLE, SA/K2/UGT and Gun/k1/Ure. p*Cpec* 7.5kbp sequence was only identified in the three koala *C. pecorum* Mc/Marsbar, DBDeUG and IpTaLE raw reads and also used in this study.

### p*Cpec* conv. PCR confirmatory assay design

Newly identified p*Cpec* sequences from a porcine L1 and three koala *C. pecorum* genomes were aligned using ClustalX. Primer pair (P frag For 5’ GTT CAC ACT CTG CCT CAT C 3’ and P frag Rev 5’ CCT ATT TAT TGG CGT CTA GG 3’) was designed to amplify a 522 bp plasmid fragment which includes a conserved intergenic region and a part of the plasmid gene CDS8. Primer sequences were tested *in silico* for secondary structures and dimerisations at Integrated DNA Technologies, Inc OligoAnalyzer 3.1 online tool (https://sg.idtdna.com/calc/analyzer). Additionally, plasmid isolation was performed from fresh available koala *C. pecorum* MC/Mars, DBDeUG, and IpTaLE cultures. Plasmid isolation and purification was performed using GeneJET Plasmid Midiprep 25 reactions, ThermoFisher Scientific, Victoria, Australia, as per manufacturer’s instructions. Freshly extracted plasmids were digested with NEB SacI (Genesearch Pty Ltd, Queensland, Australia) and visualised on the Ethidium Bromide stained 1.2 % gel, using NEB 2-Log DNA Ladder(0.1 – 10kbp) (Genesearch Pty Ltd, Queensland, Australia).

Using purified porcine L1 genomic DNA and extracted plasmid DNA from koala *C. pecorum* MC/Mars, DBDeUG, and IpTaLE as templates and positive controls (as plasmid contigs were identified in their genome sequences), conventional PCR to amplify 522 bp plasmid fragment was performed. In the same assay we used purified pig L17 and L71 genomic DNA as negative controls (as plasmid contigs were not identified in their genome sequences). The plasmid primers were additionally tested against genomic avian *C. psittaci* DNA extracted from a cultured isolate in order to test for unspecified primer binding. After successful amplification of the 522 bp plasmid fragments from templates only, the presence of the amplicon was confirmed on 1.5 % TBE gel, purified and sent off for confirmatory dideoxy sequencing.

We tested the detection limit of our p*Cpec* screening assay, based on a conventional PCR using: i) serially diluted p*Cpec* from 10^10^ to 10^1^ copies/μl as a template in triplicate; and ii) *C. pecorum* positive clinical sample Bella UGT, with 10^6^*C. pecorum* genome copy number/μl diluted to 10^1^*C. pecorum* genome copy number/μl, as a template in duplicate. The detection limit for p*Cpec* assay used in the present study was from 10^10^ to 10^2^ p*Cpec* fragment copies/μl, as determined by amplicon visualisation on 1.5 % TBE gel.

### PCR-based p*Cpec* screening

After confirming the 522 bp amplicon sequence, we applied the above described assay to screen for plasmid presence in koala and livestock *C. pecorum* samples. The full lists of screened samples are outlined in Additional file 4: Table S3 and Additional file 6: Table S4. The testing of these swab samples, collected as a part of routine diagnostic investigations of koalas and livestock, has been considered by the University of Sunshine Coast (USC) Animal Ethics Committee and the requirement for ethics approval was waived (AN/E/14/01 and AN/E/14/02). PCR assays for screening 522 bp p*Cpec* fragment were prepared to a total reaction volume of 50 μl including 1X Amplitaq Gold® 360 Master Mix (Life Technologies, Victoria, Australia), 0.3 μM of forward and reverse primer each (Integrated DNA Technologies New South Wales, Australia) and 3 μl DNA template. Negative (dH_2_0 and L17 and/or L71 DNA) and positive (MC/Marsbar plasmid DNA and L1 gDNA) controls were included in each amplification assay. The cycling conditions included an initial denaturation (10 min, 95 °C) followed by 35 cycles of denaturation (30s, 95 °C), annealing (30s, 57.5 °C) and extension (1 min, 72 °C), followed by a final extension (7 min, 72 °C). Upon amplification, PCR products were detected on a 2 % Ethidium bromide agarose gel and visualised under an UV transilluminator and purified, using a High Pure PCR product purification kit (Roche, New South Wales, Australia). A subset of 15 PCR products was directly sequenced using a BigDye Terminator v3.1 Cycle Sequencing kit (Life Technologies, Victoria, Australia) and subsequently purified according to the manufacturer’s instructions. Sequencing was performed at the Institute for Future Environments (IFE), Queensland University of Technology (QUT), Brisbane, Australia using the Applied Biosystems ABI3500 Gene analyser.

## Results and discussion

### *Porcine* C. pecorum *L1, L17 and L71 genome assemblies*

Using *C. pecorum*-specific sequence capture probes [2], we acquired whole genome sequences for three porcine *C. pecorum* isolates. *C. pecorum* strains L1 and L17 were isolated from the lungs of two different pigs presenting with pneumonia, while the L71 strain was a joint isolate from a case of polyarthritis [20, 21]. Consistent with the high average read depth (~2400X), *de novo* assembly resolved the genomes of these *C. pecorum* isolates into 3 – 5 contigs for each isolate (Table 1). Read-mapping to a reference *C. pecorum* E58 genome [15] confirmed almost whole chromosome coverage (~100 %) for all three genomes. Similar to the previously described *C. pecorum* genomes [1–3, 15], our annotation pipelines predicted ~ 1000 coding DNA sequences (CDS’s), including the three rRNA and 38 tRNA genes. The draft genome sizes of ~1.106 Mbp are almost identical to all other available *C. pecorum* genomes, with the exception of L71 which has a slightly larger draft genome size of 1.115 Mbp (Table 1).

**Table 1:** General description of pig *C. pecorum* L1, L17 and L71 samples and their genomes

	L1	L17	L71
Host disease	Pneumonia	Pneumonia	Polyarthritis
Tissue isolated from	Lung	Lung	Joint
Year of isolation	1969	1969	1969
Country	Austria	Austria	Austria
*C. pecorum* load and amount of DNA	2.15 X 10^6^ copies/μl; 1000 ng	2 X 10^6^ copies/μl; 1000 ng	1.5 X 10^6^ copies/μl; 1000 ng
Total No. of filtered reads	48 485 057	41 185 214	42 490 870
Av. filtered read length	95.1	95.4	95.1
Total No. of filtered reads for *de novo* assembly	38 807 222 (80 %)	31 115 875 (76 %)	32 594 606 (77 %)
Contigs	5 (9.7 – 623 Kbp)	3 (10–790 Kbp)	5 (11.9 –780 Kbp)
N50	623 872	293 736	780 721
Av. read depth	2569X	1800X	3020X
Genome size (bp)	1 106 140	1 106 210	1 115 012
No. of predicted CDS	1007	1002	1002
% GC	41.1 %	42 %	41.1 %
Plasmid	Yes (7.5 Kbp)	No	No
Tryptophan operon	Present (*Trp*ABFCDR, *kyn*U, *prs*A)	Present (*Trp*ABFCDR, *kyn*U, *prs*A)	Present (*Trp*ABFCDR, *kyn*U, *prs*A)
Biotin and pyrimidine genes	Present (*bio*BFDA; *pyr*EHG, *ndk*)	Present (*bio*BFDA; *pyr*EHG, ndk)	Present (*bio*BFDA; *pyr*EHG, *ndk*)
Accession numbers	LFRH01000000	LFRK01000000	LFRL01000000

Interestingly, we also identified a distinct 7.5kbp contig (with 153X coverage), assembled only from *C. pecorum* L1 strain reads. BLAST searches revealed that this contig shared 70 % sequence identity to other related chlamydial species plasmids and 99 % sequence identity to a 710 bp sequence previously identified from a suspected *C. pecorum* plasmid (accession number M32752.1) [13], based on blastn and discontiguous megablast BLAST searches (Additional file 1: Table S1). The annotation of this L1 7.5kbp contig resulted in eight CDSs specific for chlamydial plasmids.

Sequence capture methodology continues to be an effective tool for acquiring high quality whole genome sequence data [2, 38, 39], especially when cultured isolate gDNA of high concentration and genome copy number is used as a template, as in the present study. High read depths, complete genome coverage, and sequence homogeneity for our three pig strains was not surprising, as they were all pure cultured isolates, contrasting the sequence heterogeneity previously seen in *C. pecorum*-positive clinical swab studies [2]. Interestingly, design of the *C. pecorum* species-specific probes [2] also allowed for identification of distinct and/or novel sequences, such as the full length plasmid contig detected in the L1 draft genome. Now that whole genome sequences for porcine *C. pecorum* are available, the use of *C. pecorum*-specific RNA probes for sequence capture of *C. pecorum* DNA can be further improved in order to reveal features not previously observed or investigated, and to improve coverage of the polymorphic genomic regions in this species.

### *Re-evaluating the genetic differences between* C. pecorum *strains*

**Fig. 1:**
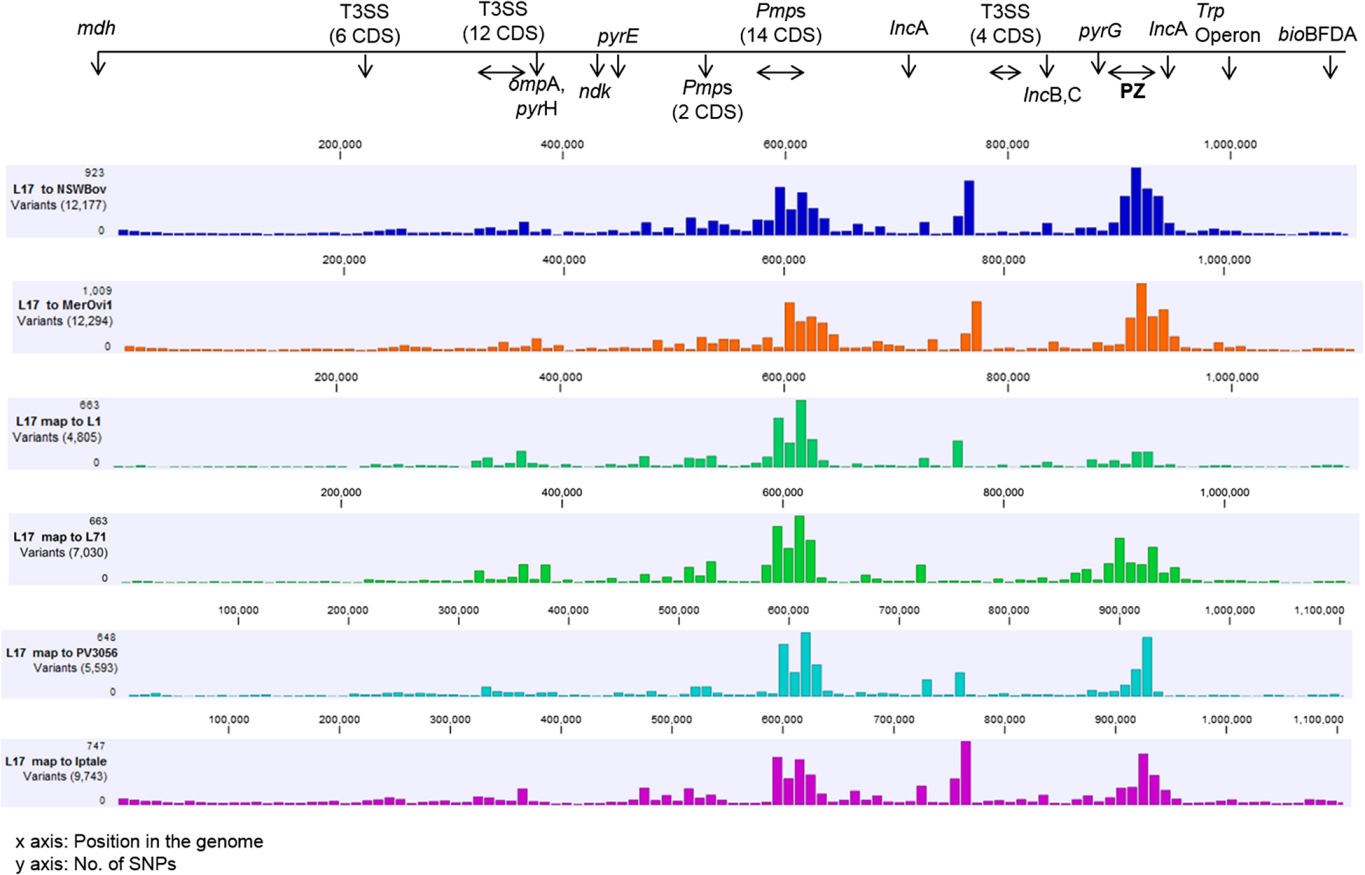
SNP distribution and identification of genomic elements across the porcine L17 genome in comparison to cattle NSW/Bov/SBE and PV3056, sheep Mer/Ovi1/Jnt, porcine L1 and L71, and koala IpTaLe genomes. All genomes start from malate dehydrogenase (*mdh*) gene. Genomic location of Type 3 Secretion System gene clusters (T3SS), major outer membrane protein (*omp*A), pyrimidine genes (*pyr*EHG, *ndk*), polymorhic membrane protein (*pmp*) gene clusters, inclusion proteins genes (*Inc*ABC), tryptophan (*Trp*ABFCDR, *kyn*U, *prs*A) and biotin operon (*bio*BFDA) genes are denoted on the horizontal line above the histograms. Variants, represented as histograms of a 10kbp size, were determined in CLC Genomics (CLCBio, Qiagen) platform

The previously described *C. pecorum* gene order and synteny [3, 15] remained conserved in the porcine *C. pecorum* genomes with nearly all variation, once again, limited to SNPs (Fig. 1). Comparison of the porcine L1, L17 and L71 genome sequences against those from other hosts revealed the most SNPs between our *C. pecorum* porcine strains and the genomes of *C. pecorum* strains previously sequenced from sheep (~11 800 SNPs; Table 2). The closest *C. pecorum* strains from other hosts, on the other hand, were from a cow (PV3056/3) and a koala (SA/K2/UGT) with approximately 5800 and 6600 SNPs, respectively. Interestingly, the majority of koala *C. pecorum* strains differed from porcine strains by ~ 9500 SNPs, less than sheep and cattle strains (Table 2). Pairwise comparison of the genome sequences revealed that the majority of SNPs were found in two major clusters, including (i) the plasticity zone (PZ); and (ii) the major polymorphic membrane (*pmp*) gene cluster (Fig. 1).

**Table 2:** The number of nucleotide differences between porcine *C. pecorum* genomes when mapped to a reference porcine, sheep, cattle, and koala *C. pecorum* genome

Mapped to Reference	Porcine L1	Porcine L17	Porcine L71
Porcine L1	–	4 376	6 665
Porcine L17	4 805	–	7 030
Porcine L71	6926	6276	–
Cattle PV3056/3	4 924	5 593	7 199
Koala Sa/K2/UGT	6321	6533	7215
Sheep W73	11 514	12 682	11 957
Sheep IPA	11 386	12 695	11 939
Sheep Mer/Ovi1/Jnt	10 850	12 294	11 502
Sheep Nar/S22/Rec	11 986	12 533	11 347
Cattle NSW/Bov/SBE	10 798	12 177	11 457
Koala Mc/Marsbar	8 235	9 482	9 916
Koala IpTaLE	8 565	9 743	10 171

Not surprisingly, averaging at ~600 SNPs/10Kbp, the *pmp* gene regions were a major contributor of single nucleotide differences (Fig. 1, Additional file 2: Table S2). Overall sequence similarity of different *C. pecorum pmp* gene families from the porcine, sheep, cattle, and koala strains ranged from 80 – 100 % (Additional file 2: Table S2). The Bayesian phylogenetic analyses resolved the *pmp* A, B, E, E’, D, and H gene families in clades respective to their designated gene family, while the *pmp*G family was the most divergent, resolving each of the eight *pmp*G subtypes into its own diverse clade (Additional file 3: Figure S1, A-B). Porcine *C. pecorum* L1, L17, and L71 strains had an identical genomic organisation, subtype and number of predicted *pmp* genes (*n* = 15) as the previously characterised sheep, cattle, and koala strains [1, 3] (Additional file 3: Figure S1, A-B; Additional file 2: Table S2). Sequence analyses of each of the 15 *pmp* genes from the porcine *C. pecorum* strains, indicated that only *pmp*G1 and *pmp*G9 type appear to be under positive selection pressure, with observed dn/ds ratios of 1.49, and 1.63, respectively (Additional file 2: Table S2). Subsequent sequence analyses of the *pmp*G1, and *pmp*G9 from all 16 *C. pecorum* strains analysed in this study, also returned dn/ds ratios > 1. Interestingly, *pmp* G1 and G9 phylogenies were similar to the phylogenetic relationships constructed from the core genomes of the 16 *C. pecorum* strains analysed in this study (described below), as well as the previous study [2] (Additional file 3: Figure S1,C-D).

In the recent sheep, cattle and koala *C. pecorum* comparative genomic study [1], the *pmp*G region was also identified as the most diverse genomic region, however, most of the genetic variation observed resulted in synonymous substitutions. In our study, variation in the *pmp* genes also mainly resulted in synonymous SNPs, with the exception of *C. pecorum pmp* genes G1 and G9, which appeared to be under positive selection. Predicted to have a major role in chlamydial infection due to their adhesive function in the interaction with host [40, 41], *pmp* genes are predicted to maintain some hypervariability in order to evade immune defences [42, 43]. Rapid diversification and evolution of the *pmp*G genes family has been previously observed in the genomic studies of the related ovine pathogen *C. abortus* [44], and avian pathogen *C. psittaci*, where it has been suggested that these genes may play a role in adaptation to different hosts and environments [45, 46]. The observed positive selection on these genes in the *C. pecorum* genomes may support a role in host adaptation for this pathogen, an aspect that may warrant extended investigation.

While the remaining genetic variation was evenly distributed across the porcine *C. pecorum* chromosomes, we identified additional eight genes under positive selection in the polymorphic regions. As outlined in Additional file 4: Table S3, *cp*L1_0338 and *cp*L1_0441, were both identified as effectors of the chlamydial Type 3 Secretion System (T3SS), a system of structural, chaperone and secreted effector proteins with its main function in host cell manipulation and subversion of the host cellular processes [47, 48]. The *cp*L1_0338 was predicted to be orthologous to a *C. psittaci* secreted T3SS protein (SINC) [49], while the *cp*L1_0441 was a well-studied chlamydial T3SS translocated actin-recruiting protein (*Tar*p) homologue [50]. In the porcine *C. pecorum* genomes, although polymorphic, the remaining T3SS genes were under negative selection. Positive selection on the *C. pecorum Tarp* homologue is perhaps not surprising, as the chlamydial *Tarp* was previously recognised as an important antigenic protein [51, 52]. Further, genomic studies of the *C. psittaci* and *C. trachomatis* revealed that the variation in the T3SS effector genes (such as *Tarp*) could also contribute to the differences in virulence, and host and/or tissue tropism associated with these pathogens [46, 53]. Presently the exact role of T3SS effectors in *C. pecorum* infections remains largely unknown, and will require more comprehensive *in silico* as well as cell biology analyses comprising of isolates from healthy, as well as diseased hosts to fully dissect T3SS effector roles. The remaining genes under positive selection were: (i) *cp*L1_0291, identified as a homologue of a previously described *C. pecorum* surface binding protein gene (*Srp*A), also under positive selection in koala strains [1]; (ii) *cp*L1_0675, a homologue of a macro domain (ADP-ribose binding) protein; and (iii) four genes encoded conserved chlamydial hypothetical proteins of unknown function (Additional file 4: Table S3).

### *Variation in the* C. pecorum *PZ*

**Fig. 2:**
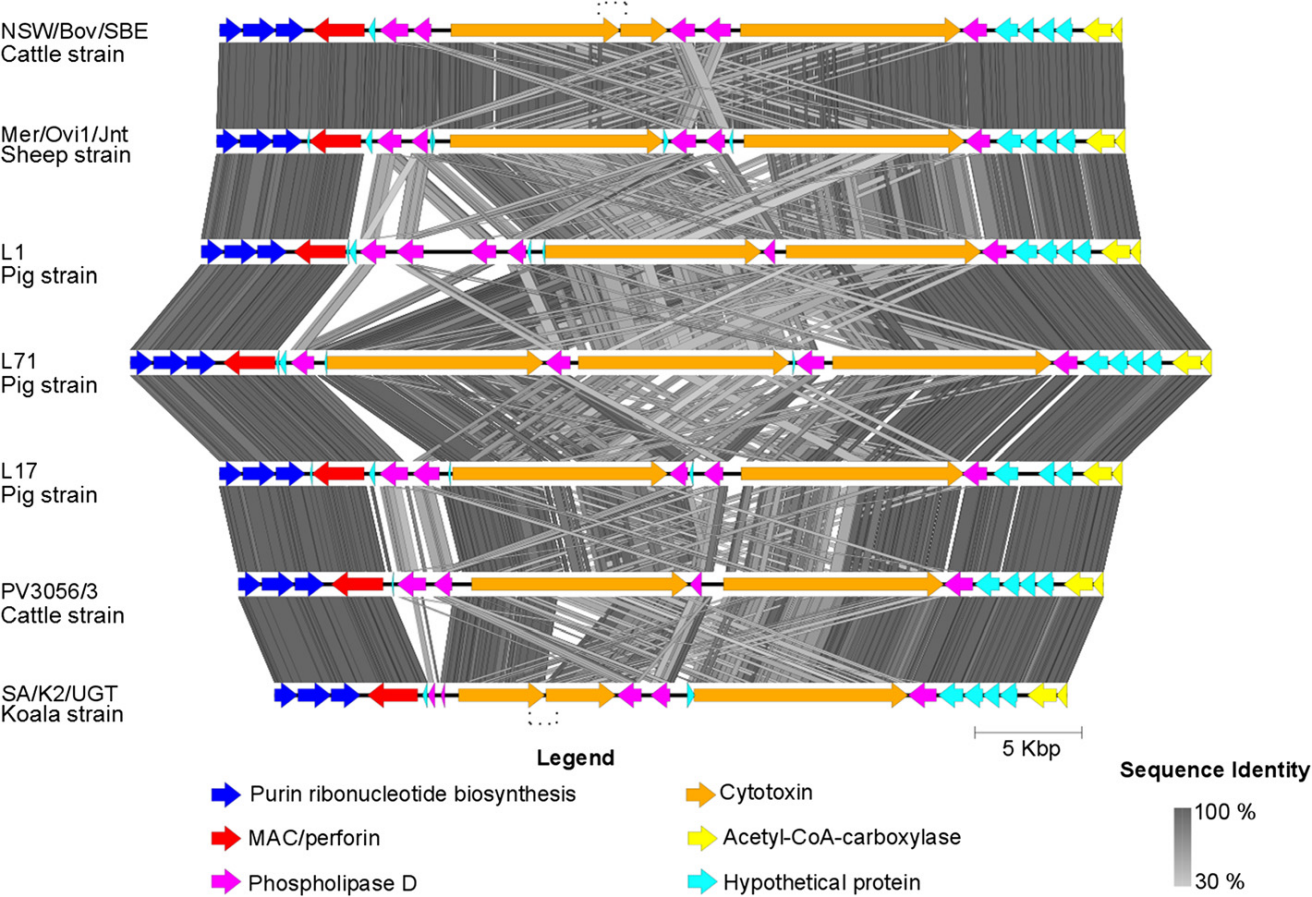
Graphical representation of the *C. pecorum* cattle NSW/Bov/SBE and PV3056, sheep Mer/Ovi1/Jnt, pigs L1, L17 and L71, and koala SA/K2/Ugt plasticity zones (PZs) tblastX comparisons. The coloured arrows represent PZ genes according to their function, as outlined in the legend. The intensity of grey shaded areas corresponds to the sequence identity. The image was generated with Easyfig [34]

In addition to the analysed *pmp* genes, the PZ, spanning from inosine-5'-monophosphate dehydrogenase (*impd*) to acetyl-CoA carboxylase (*acc*B), also displayed significant variability (Fig. 1). The PZ is a region of focus in chlamydial comparative genomic studies due to the presence and/or absence of a range of established chlamydial virulence factors [54–56]. In our study, like other *C. pecorum* strains [1–3], the porcine *C. pecorum* PZs were found to contain: (i) three purine biosynthesis genes, (ii) a *MAC/Perforin*, (iii) a variable number of *phospholipase* D (PLD) genes, (iv) two copies of *cytotoxi*n genes, (v) and two *acetyl-CoA-carboxylase* genes (Fig. 2). Some notable genetic variation, however, was also observed in these porcine *C. pecorum* strains including (i) major differences in the number of PLD genes and; (ii) the presence of a third copy of the *cytotoxin* gene in the L71 strain, as summarised in Fig. 2. In terms of PLD genes, five were observed in L17, a comparable number to that identified in the genome sequences of *C. pecorum* strains from sheep (IPA, W73 and P787), koalas (MC/Marsbar, IpTaLe, DBDeUG) and cattle (E58, NSW/Bov/SBE). The previously sequenced bovine PV3056/3 isolate also had four PLDs like L71, while the porcine L1 isolate is presently the only *C. pecorum* strain with six PLDs (Fig. 2). The variable number of PLDs is not unique to *C. pecorum* [3], as related species such as *C. trachomatis* and *C. muridarum* have also been shown to have a variable number of PLD genes among strains [55]. It has been suggested that the number of PLD genes could influence virulence [56], but whether this is the case for *C. pecorum* is still unclear, as the majority of the sequenced *C. pecorum* were sampled from hosts with evidence of chlamydial disease.

In terms of *cytotoxin* genes, almost all of the analysed strains had two copies of the *cytotoxin* gene, with observed *tox*A truncation in *C. pecorum* cattle NSW/Bov/SBE and koala SA/K2/UGT strains (Fig. 2). The porcine L71 strain however, had three copies of the *cytotoxin* gene (*tox*A: cpL71_0929, *tox*B: cpL71_0933, *tox*C: cpL71_0931) (Fig. 2). Among the genus *Chlamydia*, only *C. muridarum* has three copies of the *cytotoxin* gene [55]. Sequences from this latter species were included as an out-group in the *tox* phylogenetic tree to investigate whether the third *tox* copy identified in the L71 is similar to the *C. muridarum* third *tox* copy (Additional file 5: Figure S2). Analyses of the *C. pecorum cytotoxin* genes revealed that L71 *tox*C is more similar to *C. pecorum tox*A (69 % sequence similarity) than *tox*B (50.5 % sequence similarity). A mid-point rooted Bayesian phylogenetic tree of 11 *C. pecorum tox*A and *tox*B sequences with *tox* sequences from L71 and *C. muridarum* further revealed that the L71 *tox*C may be a duplication of the *C. pecorum tox*A, as evidenced by the clustering of this gene with *C. muridarum tox*1 and other *C. pecorum tox*A genes.

To see if *tox*C could be found in strains from other hosts, we designed a *tox*C-specific PCR (amplifying a 644 bp *tox*C specific fragment) to screen *C. pecorum* strains from pigs, sheep, cattle, and koalas (Additional file 4: Table S3). This *toxC* fragment was detected only in three more isolates: porcine pneumonia L39, L40 and HsLuRz (Additional file 4: Table S3). These strains were isolated from the same chlamydial outbreak in 1969 among Austrian pigs as the L1, L17 and L71 strains and the remaining five porcine strains (outlined in Additional file 4: Table S3) [20, 21]. Thus, based on our genomic and preliminary *tox*C analyses, it appears that the 1969 *C. pecorum* outbreak among Austrian pigs was polyclonal, with strains involved having two or three *cytotoxin* genes. Variability in the number of *tox* genes and our previously noted truncation of certain *tox* genes in some *C. pecorum* strains [1], raises the question over the function and impact of this gene in *C. pecorum* virulence.

### *Expansion on* C. pecorum *phylogenetic relationships*

**Fig. 3:**
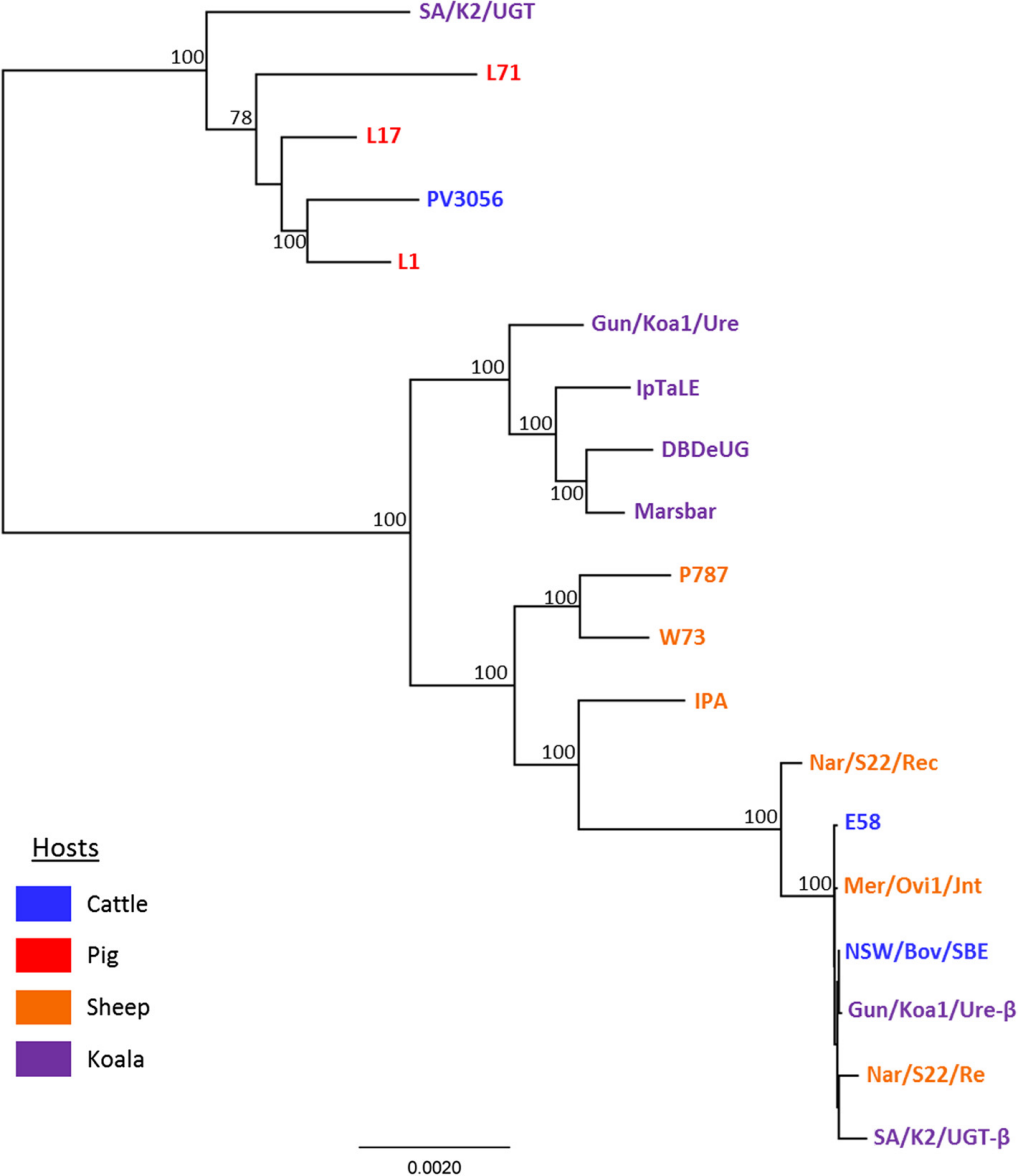
A mid-point rooted phylogenetic tree, constructed from the 280kbp core genome alignment with PhyML 3.1 using the GTR model, using 19 *C. pecorum* pig, cattle, sheep and koala strains. Bootstrap values were calculated using 500 replicates. Bootstrap values > 75 are displayed on the tree nodes. Hosts are indicated by the colouring in the legend

Using a *C. pecorum* Multi Locus Sequence Typing (MLST) scheme [23], we recently showed that porcine *C. pecorum* strains clustered in a clade separate to that of *C. pecorum* strains from a diverse range of hosts including sheep, koalas, and deer [16]. In the current study, core genome alignment, including all 19 available *C. pecorum* sequences, resolved six clades in the maximum likelihood tree (Fig. 3). The three porcine *C. pecorum* strains clustered with cattle PV3056 and a koala SA/K2/UGT strains, forming the first well supported clade (Fig. 3). Four primary koala strains resolved into their own exclusive second clade, while the third clade consisted of the European sheep *C. pecorum* isolates W73 and P787. USA polyarthritis *C. pecorum* IPA isolate formed its own fourth clade, as did the Australian sheep rectal Nar/S22/Rec strain. The final clade mainly consisted of Australian cattle and sheep strains, along with two koala *C. pecorum* strains (SA/K2/UGT-ß and Gun/Koa1/Ure-ß) detected as mixed infections with genetically distinct *C. pecorum* strains [2], and the USA cattle encephalomyelitis E58 isolate (Fig. 3).

### *Sequence-based detection of the* C. pecorum *plasmid*

**Fig. 4:**
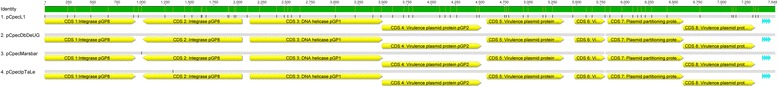
Sequence alignment of the annotated characterised p*Cpec*s from a pig and three koala *C. pecorum* strains. SNPs are highlighted in black. On the identity heat map above the alignment, green denotes 100 % sequence similarity while yellow denotes between 30 % to less than 100 % sequence similarity

No plasmids were reported in any of the *C. pecorum* genomes published prior to this study [1–3]. The unexpected finding of a *C. pecorum* 7.5 kbp plasmid (p*Cpec*) in the L1 genome prompted us to re-investigate the presence of the plasmid in other sequenced livestock and koala *C. pecorum*, using the raw reads available in our collection [1, 2]. Interestingly, full length plasmid sequences were detected only in the genomic data of *C. pecorum* Mc/Marsbar, DBDeUG and IpTaLE strains from koala hosts [1]. These additional three plasmid contigs were confirmed based on BLAST homology search and read mapping to the originally identified L1 plasmid. All identified plasmids shared an identical annotation with eight CDSs and four 22 bp tandem repeats (Fig. 4), as previously described in related chlamydial plasmids [6]. Briefly, CDSs 1 (pGP8), 2 (pGP8), 3 (pGP1), and 7 (*par*A) were denoted as putative integrase, helicase, and a partioning plasmid proteins, while the CDSs 4 (pGP2), 5 (pGP3), 6 (pGP4), and 8 (pGP6) were denoted as putative chlamydia-specific plasmid virulence proteins. The 7.5 kbp p*Cpec* sequences were 99.1 % similar to each other and shared an overall sequence similarity of 67 – 70 % to other chlamydial plasmids [6, 7] (Additional file 1: Table S1). The exact function of the p*Cpec* genes and/or their predicted products will require further *in vitro* investigations.

### *Distribution of the* C. pecorum *plasmid*

To investigate the plasmid distribution in *C. pecorum* strains, we developed a specific PCR assay that targets a 522 bp p*Cpec* fragment to screen: (i) 114 *C. pecorum* PCR positive samples collected from 67 livestock (Table 3), and (ii) 113 *C. pecorum* PCR positive samples from 73 koalas from different populations (Table 4). As summarised in Table 3, 38.6 % of the livestock *C. pecorum* strains sampled had a plasmid, with the highest detection of 63.6 % in the porcine isolates (Additional file 4: Table S3 and Additional file 6: Table S4). p*Cpec* was present in 38.4 % of the sheep *C. pecorum* strains sampled, with plasmids detected in *C. pecorum* strains detected at a range of anatomical sites (Table 3, Additional file 6: Table S4). In sheep strains, we were further able to observe that (i) genetically diverse strains (as determined by previous *C. pecorum* MLST typing [24]) infecting different sites in the same host (e.g. Nar/S45/LE and Rec; Hey/S129/Eye and Rec (Additional file 6: Table S4)) can carry p*Cpec*; and (ii) both p*Cpec*-positive and p*Cpec*-negative genetically diverse strains can circulate in a single flock (e.g. Nar/S45/Eye and Rec; Nar/S25/Vag and Rec; Nar /S40/Eye, Vag and Rec; Nar/S84 Eye and Rec (Additional file 6: Table S4)).

**Table 3:** Plasmid distribution in *C. pecorum* positive clinical samples and cultured isolates from pigs, sheep and cattle

No. of hosts	No. of samples	Plasmid detection
Sheep (*N* = 44)	86	33/86 (38.4 %)
Cattle (*N* = 12)	17	4/17 (23.5 %)
Pigs (*N* = 11)	11	7/11 (63.6 %)
	Total:	
	114	44/114 (38.6 %)

**Table 4:** Plasmid distribution in *C. pecorum* positive clinical samples and cultured isolates from different koala populations across Australia

No. of hosts	No. of samples	Plasmid detection
QLD koalas (*N* = 30)	44	32/44 (72.7 %)
NSW koalas (*N* = 15)	19	16/19 (84.2 %)
Vic koalas (*N* = 9)	14	11/14 (78.5 %)
SA koalas (*N* = 19)	36	4/36 (11.1 %)
	Total:	
	113	63/113 (55.8 %)

In cattle, p*Cpec* was detected in ocular, brain, joint and faecal *C. pecorum* isolates (Table 3, Additional file 4: Table S3). p*Cpec* was less common in the limited number of clinical samples analysed (Table 3, Additional file 6: Table S4). A subset of these *C. pecorum* positive clinical samples, collected from two calves (WA/B65 and WA/B31), with sporadic bovine encephalomyelitis (SBE) were described previously in a case study [25] where *C. pecorum* MLST [23] indicated that brain, liver and lung *C. pecorum* WA/B65 and WA/B31 strains were of the same *C. pecorum* genotype (denoted ST23) as other SBE isolates such as NSW/Bov/SBE and E58. In the present study we observed that all of these samples were p*Cpec* negative, but that a genetically distinct *C. pecorum* positive ileal sample was p*Cpec* positive. p*Cpec* was similarly absent in several other *C. pecorum* SBE isolates (Additional file 4: Table S3). The absence of p*Cpec* from SBE-associated *C. pecorum* strains based on this limited p*Cpec* screening may imply that the p*Cpec* is not a virulence factor that affects SBE pathogenesis.

The plasmid was more commonly detected in the koala *C. pecorum* samples studied, with 72.7 %, 84.2 % and 78.5 % distribution in the samples collected from Queensland (QLD), New South Wales (NSW) and Victoria (Vic), respectively (Table 4). In contrast, p*Cpec* was less common in the South Australian (SA) koala samples analysed (11.1 %) (Table 4).

Together, this data indicates that the *C. pecorum* plasmid is not ubiquitous, in contrast to *C. trachomatis*, where nearly all naturally occurring strains contain the plasmid [57]. In terms of confidence in our detection, we believe that the sensitivity of our p*Cpec* assay was sufficient to detect plasmid even in low *C. pecorum* (<1x10^2^*C. pecorum* genome copies) positive samples as the chlamydial plasmid is thought to have between two and 10 copies/per chlamydial genome, based on other studies [57]. As outlined in Additional file 4: Table S3 and Additional file 6: Table S4, the number of *C. pecorum* genomes for each screened sample ranged from a 5.0x10^1^ to 9.8x10^6^*C. pecorum* genome copies/μl of the extracted DNA (as determined by the *C. pecorum*-specific qPCR screen [5]), while the tested p*Cpec* fragment lower detection limit was 1x10^2^ plasmid copies/μl. Indeed, in the present study, there was no apparent relationship between ability to detect plasmid and the number of genome copies in a sample, with plasmid detected in koala A3, R15, R1-17 UGT and sheep Cur/L236/Vag samples with only ~1x10^2^*C. pecorum* genome copies (Additional file 6: Table S4). Nevertheless, we do also acknowledge that it is possible that we may have missed detection of p*Cpec* in samples with < 1x10^2^*C. pecorum* genome copies if each *C. pecorum* genome were to be associated with only single p*Cpec* copy.

The reliance on archived samples in the present study precluded observation of trends in disease, tissue and/or host specificity for plasmid distributions; most PCR positive samples or isolates were from diseased animals, sample sizes were small, and sampling was spatially clustered (Additional file 4: Table S3 and Additional file 6: Table S4). As prevalence among such samples may not accurately reflect prevalence in wider populations and small sample sizes would confer very wide confidence intervals on prevalence estimates, it was not possible to compare prevalence across geographic locations, hosts, or disease states with any certainty. However, the markedly lower apparent prevalence of p*Cpec* in South Australian koala samples (11 %) (Table 4, Additional file 6: Table S4) relative to koala samples from QLD, Vic, and NSW (73 – 84 %) appears worthy of further investigation. Anecdotal evidence and preliminary PCR-based screening studies [58] (Speight K.N., Polkinghorne A., Penn R., Boardman W., Timms P., Fraser T., Johnson K., Faull R., Bate S., Woolford L., 2015, unpublished observations), suggest that *C. pecorum* infections in SA may be less common and pathogenic than those described in eastern and northern Australian states (QLD and NSW) [17]. Similarly low prevalence of chlamydial disease has been proposed for Victorian koalas. In other chlamydial species, there is strong evidence linking the chlamydial plasmid to pathogenic potential. For example, in the mouse model, it was demonstrated that plasmid deficient *C. trachomatis* isolates are less infective and less virulent [9]. Similarly, *C. muridarum* studies demonstrated critical role of the plasmid in the development and severity of intrauterine infections [8, 59]. Given the multifactorial nature of disease pathogenesis, however, the picture is unlikely to be simple, particularly in the koala. As such, further studies are required to examine the impact of the presence or absence of this plasmid on *C. pecorum* infection, growth and pathogenicity.

As an interesting sidenote, in *C. trachomatis*, it was also observed that the chlamydial plasmids are correlated with the accumulation of glycogen granules inside the inclusion (used as an energy source) and that some plasmid loci (like *pgp*4) may regulate transcription of the corresponding chromosomal genes involved in glycogen pathways [7, 60]. In their 1992 study [22], Kaltenboeck and Storz examined the biological properties of the same pig strains that we used for our genomic and plasmid analyses. Inclusions of L1, R106, 1710S, and 1920Brz pig strains were observed to be aberrant, coarse and patchy, with pleomorphic reticulate bodies, in contrast to other pig strains (such as L71 and L40) with regular inclusions. In our study we detected plasmid in the same strains (Additional file 4: Table S3), with plasmid fully characterised from L1, while the regular inclusion strains L71 and L40 were plasmid negative. Given the differing distribution of the *C. pecorum* plasmid and the otherwise conserved coding sequences of this plasmid relative to the plasmid in other chlamydial species, this study raises important questions over the function of the *C. pecorum* plasmid. These questions can only be answered with additional *in vitro* and *in vivo* investigations.

## Conclusion

In the present study, we sequenced three *C. pecorum* genomes from pigs presenting with pneumonia (L1 and L17 strains) and polyarthritis (L71), and compared them to genomes of other *C. pecorum* strains from sheep, cattle and koala. The main genetic differences were limited to the highly variable *pmp* region and the *C. pecorum* PZ. Besides the variable number of *phospholipase* D genes, we also observed a third full copy of the *cytotoxin* gene (*tox*C) in the PZ of the porcine L71 genome. Preliminary screening for a *tox*C fragment in other *C. pecorum* strains revealed that this additional *tox* gene could only be found in other porcine strains (L39, L40 and HsLuRz).

The most significant and novel genomic feature described in this study was the identification of the chlamydial plasmid in *C. pecorum*. Based on our PCR-based screening approach, this plasmid does not appear to be ubiquitous, raising questions over its function and impact on chlamydial pathogenesis in light of the important role it plays in other chlamydial species such as *C. muridarum* [8, 59]. It appears possible that the plasmid is less common in strains infecting koalas in South Australia, relative to those in other states. Although no association was observed between the presence or absence of the plasmid in association with tissue, host or disease in the present study, differences in the distribution of this plasmid among koala populations and individuals with different levels of chlamydial disease appears to warrant further investigations. Whether the *C. pecorum* plasmid is a virulence factor or just a “harmless accessory” otherwise, remains to be elucidated.

### Availability of supporting data

All of the available supporting data from this manuscript is provided as Additional Files.

## Additional files

Additional file 1: Table S1.
*C. pecorum* L1 plasmid sequence identity comparisons based on discontiguous megaBLAST hits. (PDF 338 kb) Additional file 2: Table S2. Sequence analyses of the porcine L1, L17, and L71 *C. pecorum pmp* gene families. (PDF 357 kb) Additional file 3: Figure S1. Bayesian phylogenetic analyses of the *C. pecorum pmp* gene subtypes. Posterior probabilities > 0.70 are displayed on the tree nodes. A: *C. pecorum pmp*s A,B, D, E, E, H gene family phylogenies from the 16 porcine, sheep, cattle, and koala *C. pecorum* strains analysed in this study; B: *C. pecorum pmp*G gene family phylogeny from the 16 porcine, sheep, cattle, and koala *C. pecorum* strains analysed in this study; C: *pmp*G1 phylogenetic tree constructed from the 2406 bp alignment of 16 porcine, sheep, cattle, and koala *C. pecorum* strains analysed in this study; and D: *pmp*G9 phylogenetic tree constructed from the 2841 bp alignment of 16 porcine, sheep, cattle, and koala *C. pecorum* strains analysed in this study. Hosts are indicated by the colouring in the legend. (PNG 300 kb) Additional file 4: Table S3. Characteristics of the polymorphic chromosomal genes under positive selection analysed in the present study from three porcine *C. pecorum* L1, L17, and L71 strains. (PDF 267 kb) Additional file 5: Figure S2. Bayesian phylogenetic analyses of the cytotoxin genes from the subset of 12 porcine, sheep, cattle, and koala *C. pecorum* strains. Posterior probabilities > 0.70 are displayed on the tree nodes, while *C. muridarum* Nigg *tox* 3 is used as an out-group. (PNG 219 kb) Additional file 6: Table S4.
*C. pecorum* isolates and clinical samples Additional file 4: Table S3 and Additional file 6: Table S4. (XLSX 23 kb)

## Abbreviations

Bp: Base pairs
CDS: Coding DNA sequence
kbp: Kilo base pairs
Mbp: Mega base pairs
MLST: Multi Locus Sequence Typing
PCR: Polymerase chain reaction
*pmp*: Polymorphic membrane protein
PZ: Plasticity zone
qPCR: Quantitative polymerase chain reaction
rRNA: Ribosomal RNA
SNP: Single nucleotide polymorphism
tRNA: Transfer RNA
