# Supplementary material for: Genetic diversity in the plasticity zone and the presence of the chlamydial plasmid differentiates Chlamydia pecorum strains from pigs, sheep, cattle, and koalas
Source: BMC Genomics. 2015 Nov 4;16:893. doi: 10.1186/s12864-015-2053-8 (PMC4632680; doi:10.1186/s12864-015-2053-8)
Supplement: Additional file 1: Table S1. — C. pecorum L1 plasmid sequence identity comparisons based on discontiguous megaBLAST hits. (PDF 338 kb) [file 12864_2015_2053_MOESM1_ESM.pdf]

Table S1. *C. pecorum* L1 plasmid sequence identity comparisons based on discontiguous megaBLAST hits.

**Identity to:**	**Plasmid size**	**Accession number**	**% sequence similarity**	**% query cover**
C. pneumoniae **pLPColN**	7530 bp	CP001714.1	71%	96%
C. psittaci **p6BC**	7533 bp	CP002550.1	70%	93%
C. trachomatis **pCTA**	7510 bp	CP000052.1	63%	67%
C. muridarum **pMoPn**	7501 bp	AE002162.1	67%	71%
C. caviae **pCpGP 1**	7966 bp	AE015926.1	70%	95%
C. avium **p10DC88**	7099 bp	CP006572.1	89%	70%
C. felis **pCfe 1**	7552 bp	AP006862.1	70%	90%
C. pecorum **DNA for cross-hybridisation**	710bp	M32752.1	99%	9%
pCpec Marsbar	7547bp	KT223775	99.1%	100%
pCpec IpTaLE	7547bp	KT223772	99.1%	100%
pCpec DbDeUg	7547bp	KT223770	99.1%	100%
