# Supplementary material for: Genetic diversity in the plasticity zone and the presence of the chlamydial plasmid differentiates Chlamydia pecorum strains from pigs, sheep, cattle, and koalas
Source: BMC Genomics. 2015 Nov 4;16:893. doi: 10.1186/s12864-015-2053-8 (PMC4632680; doi:10.1186/s12864-015-2053-8)
Supplement: Additional file 2: Table S2. — Sequence analyses of the porcine L1, L17, and L71 C. pecorum pmp gene families. (PDF 357 kb) [file 12864_2015_2053_MOESM2_ESM.pdf]

Table S2. Sequence analyses of the porcine L1, L17, and L71 *C. pecorum pmp* gene families.

**Annotation and sequence length (bp)**	**Locus tag in L1**	**Total No. of polymorhisms**	**No. of indels**	**Δnt in protein coding region***	**No. of non-synonymous substitutions**	**No. of synonymous substitutions**	**dn/ds ratio^**	**% sequence similarity between L1, L17 and L71**	**% sequence similarity between 16 *C. pecorum* strains**
**pmpD** (4215bp)	cpL1_0082	5	0	5	2	3	0.211	99.9 - 100	99.3 -100
**pmpB** (5091bp)	cpL1_0475	291	30	260	88	172	0.168	95.2 -98.2	89.6 - 100†
**pmpA** (2844bp)	cpL1_0476	29	0	29	13	16	0.256	99.1 – 99.5	98.8 - 100
**pmpE#** (1992bp)	cpL1_0540	211	3	177	69	108	0.229	92.2 – 94.7	95.3 - 100†
**pmpE'** (2907 bp)	cpL1_0541	573	54	486	236	250	0.309	83.0 – 91.1	80.0 - 100
**pmpH** (3021bp)	cpL1_0542	209	15	193	68	125	0.181	94.7 – 95.7	94.4 - 100
**pmpG1** (3045bp)	cpL1_0543	233	52	174	119	55	1.49	94.0 – 96.3	87.5 - 100†
**pmpG2** (2751bp)	cpL1_0544	41	0	41	18	23	0.870	98.8 – 99.2	98.8 – 100
**pmpG3** (2460bp)	cpL1_0547	34	0	34	5	29	0.052	98.7 – 99.8	98.5 - 100
**pmpG4** (3207bp)	cpL1_0548	264	42	222	93	129	0.181	93.4 – 95.9	92.9 - 100
**pmpG5#** (1692bp)	cpL1_0550	378	66	334	163	171	0.294	81.0 – 86.3	n.a
**pmpG6** (2826bp)	cpL1_0551	275	36	218	96	122	0.386	92.4 – 94.6	92.3 - 100
**pmpG7** (2898 bp)	cpL1_0552	231	21	206	98	108	0.287	93.8 – 95.5	92.1 - 100
**pmpG8#** (2616bp)	cpL1_0553	136	21	115	57	58	0.338	92.6 – 96.5	92.3 - 100†
**pmpG9** (2841bp)	cpL1_0940	18	0	18	15	3	1.63	99.5 – 99.8	99.3 - 100

#: partial sequence analysed, including autotransporter (AT) domain of the gene; *: Δnt (No. of polymorphic sites), excluding gaps in the alignment; ^: ratio of the average number of non-synonymous substitutions per non-synonymous site and synonymous substitutions per synonymous site (Jukes - Cantor corrected), estimated excluding gaps in the alignment; †: partial sequence alignment, including middle and AT domain of the gene; n.a: not applicable for all due to contigs breaks.
