# Supplementary figures and images for: Genetic diversity in the plasticity zone and the presence of the chlamydial plasmid differentiates Chlamydia pecorum strains from pigs, sheep, cattle, and koalas
Source: BMC Genomics. 2015 Nov 4;16:893. doi: 10.1186/s12864-015-2053-8 (PMC4632680; doi:10.1186/s12864-015-2053-8)

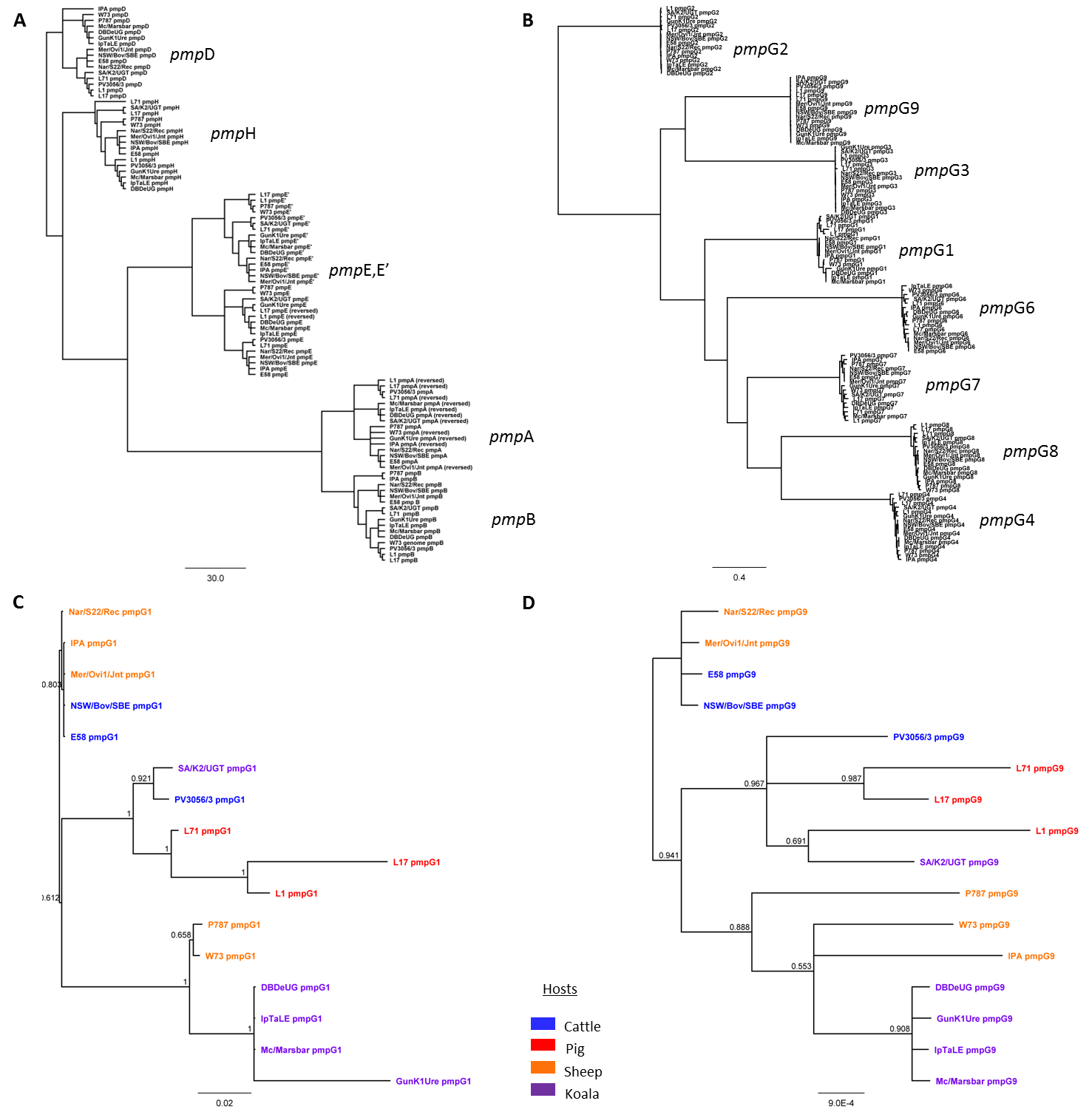

Supplement: Additional file 3: Figure S1. — Bayesian phylogenetic analyses of the C. pecorum pmp gene subtypes. Posterior probabilities > 0.70 are displayed on the tree nodes. A: C. pecorum pmps A,B, D, E, E, H gene family phylogenies from the 16 porcine, sheep, cattle, and koala C. pecorum strains analysed in this study; B: C. pecorum pmpG gene family phylogeny from the 16 porcine, sheep, cattle, and koala C. pecorum strains analysed in this study; C: pmpG1 phylogenetic tree constructed from the 2406 bp alignment of 16 porcine, sheep, cattle, and koala C. pecorum strains analysed in this study; and D: pmpG9 phylogenetic tree constructed from the 2841 bp alignment of 16 porcine, sheep, cattle, and koala C. pecorum strains analysed in this study. Hosts are indicated by the colouring in the legend. (PNG 300 kb) [file 12864_2015_2053_MOESM3_ESM.png]

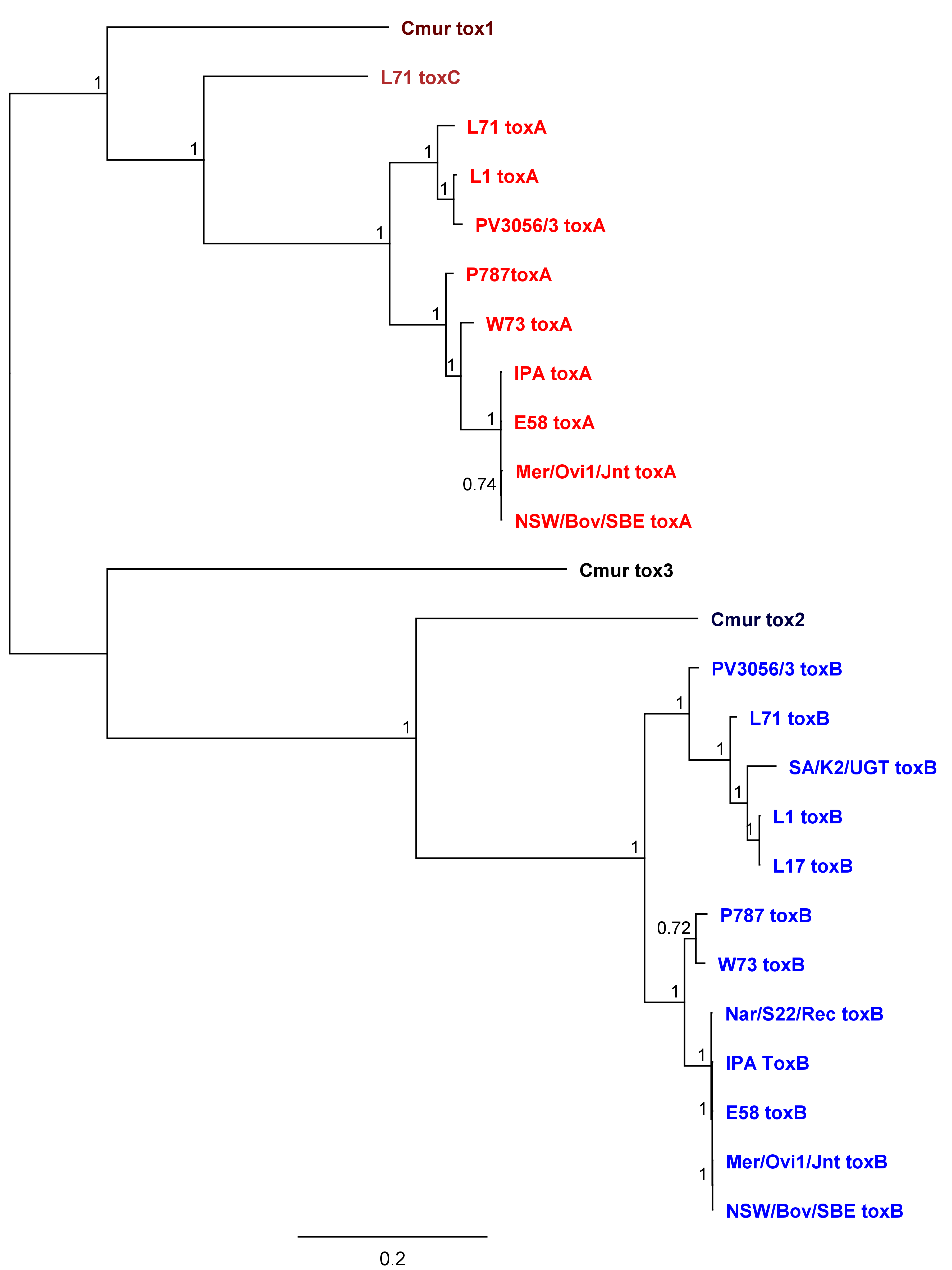

Supplement: Additional file 5: Figure S2. — Bayesian phylogenetic analyses of the cytotoxin genes from the subset of 12 porcine, sheep, cattle, and koala C. pecorum strains. Posterior probabilities > 0.70 are displayed on the tree nodes, while C. muridarum Nigg tox 3 is used as an out-group. (PNG 219 kb) [file 12864_2015_2053_MOESM5_ESM.png]
