# Supplementary material for: Genetic diversity in the plasticity zone and the presence of the chlamydial plasmid differentiates Chlamydia pecorum strains from pigs, sheep, cattle, and koalas
Source: BMC Genomics. 2015 Nov 4;16:893. doi: 10.1186/s12864-015-2053-8 (PMC4632680; doi:10.1186/s12864-015-2053-8)
Supplement: Additional file 4: Table S3. — Characteristics of the polymorphic chromosomal genes under positive selection analysed in the present study from three porcine C. pecorum L1, L17, and L71 strains. (PDF 267 kb) [file 12864_2015_2053_MOESM4_ESM.pdf]

Table S3. Characteristics of the polymorphic chromosomal genes under positive selection analysed in the present study from three porcine *C. pecorum* L1, L17, and L71 strains

**Annotation and sequence length (bp)**	**Locus tag in L1**	**Predicted function/product***	**Δnt in protein coding region***	**No. of non-synonymous substitutions**	**No. of synonymous substitutions**	**dn/ds ratio^**	**Base coordinates in L1**
**Hypothetical protein** (1912 bp)	cpL1_0291	*C. pneumoniae* 76kDa orthologue/wall surface anchor SrpA1 protein	159	87	72	1.11	327 – 332kbp
**Conserved hypothetical protein** (1089 bp)	cpL1_0338	Chlamydial T3SS effector protein/*C. psittaci* SINC orthologue	174	107	67	1.30	381.5 – 383.5kbp
**Conserved hypothetical protein** (2406 bp)	cpL1_0441	T3SS effector *Tar*p homologue	105	56	49	1.11	475.5 – 478.5kbp
**Conserved hypothetical protein** (1683 bp)	cpL1_0538	Chlamydial conserved protein	517	324	193	1.88	587 – 591.5kbp
**Conserved hypothetical protein** (2246 bp)	cpL1_0539	Chlamydial conserved protein/transcriptional regulator	575	385	190	1.33	587 – 591.5kbp
**Conserved hypothetical protein** (1460 bp)	cpL1_0645	Chlamydial conserved protein	61	39	22	1.66	725 – 729.5kbp
**Conserved hypothetical protein** (1794 bp)	cpL1_0646	Chlamydial conserved protein	101	75	26	1.56	725 – 729.5kbp
**Macro domain protein** (1140 bp)	cpL1_0675	ADP-ribose binding domain	189	122	67	1.72	756.5 – 758.5kbp

*: function predicted based on blastn and tblastx comparison; *: Δnt (No. of polymorphic sites), excluding gaps in the alignment; ^: ratio of the average number of non-synonymous substitutions per non-synonymous site and synonymous substitutions per synonymous site (Jukes - Cantor corrected), estimated excluding gaps in the alignment.
